# Catecholaminergic depletion within the prelimbic medial prefrontal cortex enhances latent inhibition

**DOI:** 10.1016/j.neuroscience.2010.06.066

**Published:** 2010-09-29

**Authors:** A.J.D. Nelson, K.E. Thur, C.A. Marsden, H.J. Cassaday

**Affiliations:** Schools of Psychology and Biomedical Sciences, University of Nottingham, Nottingham NG7 2RD, UK

**Keywords:** latent inhibition, prelimbic cortex, infralimbic cortex, dopamine, schizophrenia, BLA, basolateral amygdala, CPu, caudate putamen, CS, conditioned stimulus, DA, dopamine, HPLC-ECD, high performance liquid chromatography with electrochemical detection, IL, infralimbic cortex, LI, latent inhibition, mPFC, medial prefrontal cortex, NA, noradrenaline, NAc, nucleus accumbens, NPE, non-pre-exposed, OFC, orbitofrontal cortex, PE, pre-exposed, PL, prelimbic cortex, UCS, unconditioned stimulus, 5-HT, serotonin, 6-OHDA, 6-hydroxydopamine

## Abstract

Latent inhibition (LI) refers to the reduction in conditioning to a stimulus that has received repeated non-reinforced pre-exposure. Investigations into the neural substrates of LI have focused on the nucleus accumbens (NAc) and its inputs from the hippocampal formation and adjacent cortical areas. Previous work has suggested that lesions to the medial prefrontal cortex (mPFC), another major source of input to the NAc, do not disrupt LI. However, a failure to observe disrupted LI does not preclude the possibility that a particular brain region is involved in the expression of LI. Moreover, the mPFC is a heterogeneous structure and there has been no investigation of a possible role of different regions within the mPFC in regulating LI under conditions that prevent LI in controls. Here, we tested whether 6-hydroxydopamine (6-OHDA)-induced lesions of dopamine (DA) terminals within the prelimbic (PL) and infralimbic (IL) mPFC would lead to the emergence of LI under conditions that do produce LI in controls (weak pre-exposure). LI was measured in a thirst motivated conditioned emotional response procedure with 10 pre-exposures to a noise conditioned stimulus (CS) and two conditioning trials. Sham-operated and IL-lesioned animals did not show LI and conditioned to the pre-exposed CS at comparable levels to the non-pre-exposed controls. 6-OHDA lesions to the PL, however, produced potentiation of LI. These results provide the first demonstration that the PL mPFC is a component of the neural circuitry underpinning LI.

Latent inhibition (LI) manifests as poorer conditioning to a stimulus that has been previously presented without consequence (Lubow and Moore, 1959). In terms of the psychological processes underlying LI, it is believed that reduced salience of the stimulus in consequence of the previous exposure without consequence interferes with learning and/or performance of the conditioned response (Lubow and Weiner, 2010). As salience processing and attentional processes are dysfunctional in schizophrenia, the neural substrates of LI have received considerable attention in the last two decades.

LI is disrupted by amphetamine in both rats (Solomon et al., 1981; Weiner et al., 1984) and humans (Gray et al., 1992; Kumari et al., 1999) and is absent in acutely ill schizophrenia patients (Baruch et al., 1988) but is potentiated by antipsychotics (APD) in both rats (Weiner and Feldon, 1987; Shadach et al., 2000) and humans (Williams et al., 1997). Consequently, LI has received attention as a putative animal model of cognitive deficits in schizophrenia (e.g. Weiner, 1990, 2003; Gray et al., 1991; Weiner and Arad, 2009; for discussion of alternative models see, e.g. Lipska and Weinberger, 2000; Geyer and Moghaddam, 2002).

Investigations into the neuroanatomical substrates of LI have shown that the nucleus accumbens (NAc) and regions with afferent connections to NAc—entorhinal cortex, hippocampus, orbitofrontal cortex (OFC) and basolateral amygdala (BLA)—play a key role in regulating the expression of LI (e.g. Honey and Good, 1993; Tai et al., 1995; Coutureau et al., 1999; Schiller and Weiner, 2004; Gal et al., 2005). However, both electrolytic lesions to the medial prefrontal cortex (mPFC) and its subregions as well as excitotoxic lesions to the entire mPFC have been shown to spare LI (Joel et al., 1997; Lacroix et al., 1998, 2000a). The failure to observe effects on LI after manipulations to the mPFC is surprising in view of considerable evidence implicating frontal dysfunction in the psychopathophysiology of schizophrenia (e.g. Andreasen et al., 1992; Goodman et al., 1999; Barch et al., 2001; MacDonald et al., 2005).

However, the failure of mPFC lesions to disrupt LI does not preclude the possibility that the mPFC is involved in the expression of LI as the role of a specific brain region in LI may only emerge under conditions that do not produce LI in controls (Weiner and Arad, 2009). For example, lesions to the BLA or entire NAc do not disrupt LI under conditions that produce LI in sham-operated controls (low number of conditioning trials and high number of stimulus pre-exposures) but lead to the emergence of LI under conditions (high number of conditioning trials or low number of stimulus pre-exposures) that do not yield LI in controls (e.g. Gal et al., 2005; Schiller and Weiner, 2005). One previous study has examined the potential involvement of the mPFC in the expression of LI under conditions that do not yield LI in controls (high number of conditioning trials) and found no effects of mPFC lesions (Schiller and Weiner, 2004). However, the mPFC is both anatomically and functionally heterogeneous and there has been no investigation of the involvement of the prelimbic (PL) and infralimbic (IL) subregions of the mPFC in mediating the expression of LI using experimental parameters that do not lead to the emergence of LI in controls.

Thus to dissociate the potential role of different neuroanatomical systems within the mPFC, we tested the effects of 6-hydroxydopamine (6-OHDA) lesions to dopaminergic fibres within the PL and IL cortices on LI using experimental parameters explicitly designed not to produce LI in controls (low number of stimulus pre-exposure).

## Experimental procedures

### Subjects

The subjects were 60 adult male Wistar rats (Charles River, UK) and were caged in pairs on a 12:12 h light/dark cycle with food and water *ad libitum*. Rats were handled for approximately 10 min per day for 1 week and then at mean weight 265 g (range 225–307 g) underwent surgery. Twenty rats were randomly allocated to each of the PL and IL groups and a total of 20 rats were allocated to the sham condition (10 rats were sham operated at the PL coordinates and 10 rats were sham operated at the IL coordinates).

All procedures were carried out in accordance with the United Kingdom (UK) Animals Scientific Procedures Act 1986, Project Licence number: PPL 40/3163. The UK Act ensures full compliance with the “Principles of laboratory animal care” (NIH publication No. 86-23, revised 1985).

### Stereotaxic infusion of 6-OHDA

In order to protect noradrenergic terminals, animals received subcutaneous administration of the noradrenaline (NA) reuptake inhibitor desipramine (20 mg/kg) 40 min prior to surgery. Anaesthesia was induced by isoflurane (4%) in a N_2_O/O_2_ (1:2, v/v) mixture and maintained thereafter with isoflurane (1–2%). Stereotaxic surgery was conducted with the incisor bar set at −3.3 mm below the intra-aural line. The bone above the mPFC was removed and the dura was cut to expose the cortex. Rats received bilateral infusions of 6-OHDA or vehicle into either PL or IL mPFC at the following stereotaxic coordinates prelimbic: AP +3.8 mm; ML ±0.6 mm; DV −3.8 mm; AP +3.2 mm; ML ±0.6 mm; DV −3.6 mm; AP +2.5 mm; ML ±0.6 mm; DV −3.4 mm; infralimbic: AP +3.0 mm; ML ±0.7 mm; DV −5.4 mm (Paxinos and Watson, 2005). DV coordinates were taken from dura. Infusions were made via a 31 gauge stainless steel injector attached by polythene tubing to a 1 μl Hamilton syringe. 6-OHDA hydrobromide (24 mg/mL as salt dissolved in vehicle; Sigma, UK) or vehicle (0.9% saline/ascorbic acid 0.01% w/v) was infused manually over 2 min bilaterally in a volume of 0.2 μl per injection site. The injectors were left *in situ* for 5 min to allow absorption of the bolus and to minimize spread of the toxin. Rimadyl (0.03 ml s.c.) provided post-operative analgesia. Animals were allowed a minimum of 7 days recovery before the commencement of behavioral testing.

### Quantification of 6-OHDA lesion by HPLC-ECD

In order to quantify the degree of dopaminergic deafferentation, we used micropunch to take samples from the target structures (PL and IL) and other brain regions core NAc, shell NAc, OFC, caudate putamen (CPu), amygdala for subsequent assessment of neurotransmitter levels dopamine (DA), noradrenaline (NA) and serotonin (5-HT) by high pressure liquid chromatography with electrochemical detection (HPLC-ECD). These control regions were selected on the basis of their known connectivity with the mPFC (e.g. Sesack et al., 1989; Takagishi and Chiba, 1991) and demonstrated involvement in LI (Weiner, 2003).

Following the completion of behavioral testing, the rats were humanely killed by dislocation of the neck and decapitated. The dissection and micropunch technique used was as described by Peleg-Raibstein et al. (2004). The brains were removed rapidly and were placed ventral side up in an ice-chilled rat brain matrix (Harvard Instruments, USA). Using ice-chilled razor blades, three 2-mm coronal brain sections were cut. The posterior side of the slices corresponded to approximately +3, +1 and −3 from Bregma according to the atlas of Paxinos and Watson (2005). The brain samples were then immediately frozen on dry ice and stored at −80 °C. Subsequently, the three 2 mm coronal sections were placed posterior side up onto an ice-chilled plate. From the first section (+3 mm bregma) a 0.84 mm diameter stainless steel micropunch was used to remove the PL, ILF and OFC. From the second section (+1 bregma), the 0.84 mm diameter stainless steel micropunch was also used to remove samples of tissue from core NAc and medial shell NAc and a 1.6 mm diameter stainless steel micropunch was used to remove sample tissue from the CPu. From the third section (−3 mm bregma) a 1.6 mm diameter stainless steel micropunch was used to remove the amygdala. In each case, one punch was used per brain hemisphere. Tissue punch samples were stored in 1.5 ml Eppendorf tubes and frozen at −80 °C.

Neurotransmitter levels in the samples were determined by HPLC-ECD. The tissue samples were homogenized in 0.1 M PCA solution by sonication and centrifuged at 17400 g for 20 min at 4 °C. Neurotransmitter levels were detected using a glassy carbon flow cell (VT-03 Antec) with an Ag/AgCl reference electrode. An external standard consisting of DA, NA, 5-HT and metabolites in concentrations of 10^−7^, 0.5×10^−7^ and 10^−8^ M was injected at a volume of 4 μl for calibration. Samples were injected onto the column at 4 μl volumes, except for PL, IL, OFC and amygdala samples which were injected at 8 μl because of the higher detection thresholds in these regions.

Results were analysed using Alexys software data system. Bradford assay was used to adjust for protein content using the pellet remaining after sample centrifugation.

### Latent inhibition

#### Apparatus

Six identical fully automated conditioning chambers, housed within sound-attenuating cases containing ventilation fans (Cambridge Cognition, Cambridge, UK), were used. Each of the inner conditioning chambers consisted of a plain steel box (25 cm×25 cm×22 cm high) with a Plexiglas door (19 cm×27 cm) at the front. The floor was a shock grid with steel bars 1 cm apart and 1 cm above the lip of a 7 cm deep sawdust tray. A waterspout was mounted on one wall. The spout was 5 cm above the floor and connected to a lickometer supplied by a pump. Licks were registered a break in the photo beam within the spout, which also triggered water delivery of 0.05 ml per lick. The waterspout was illuminated when water was available. A loudspeaker for the presentation of auditory stimuli was set in the roof. A 5 s mixed frequency noise set at 85 dB (including background) served as the conditioned stimulus (CS). Footshock of 1 s duration and 1 mA intensity provided the unconditioned stimulus (UCS). This was delivered through the grid floor by a constant current shock generator (pulsed voltage: output square wave 10 ms on, 80 ms off, 370 V peak under no load conditions, MISAC Systems, Newbury, UK). Stimulus control and data collection was by an Acorn Archimedes RISC computer programmed in Basic with additional interfacing using an Arachnid extension (Cambridge Cognition).

#### Procedure

Water deprivation was introduced 1 day prior to shaping. Thereafter, the animals received 1 h and 15 min of *ad libitum* access to water in their home cage in addition to water in the experimental chambers. The stages of the conditioned emotional response (CER) procedure used in Experiment 1 were as follows:

#### Pre-training

In order to initiate licking behavior, rats were placed in the experimental chambers with their respective cage mate and were shaped for 1 day until all drank from the waterspout. No data were recorded. Thereafter, animals were individually assigned to a conditioning box for the duration of the experiment (counterbalanced by experimental group).

There then followed 5 days of pre-training, in which rats drank in the experimental chamber for 15 min each day (timed from first lick). The drinking spout was illuminated throughout, but no other stimuli were presented in this phase. Latency to first lick and total number of licks were recorded to assess any pre-existing differences in drinking (prior to conditioning).

#### Pre-exposure

Animals were placed in the chambers where the pre-exposed animals received 10 5 s CS presentations with an average inter-stimulus interval of 60 s. The non-pre-exposed control animals were confined to the chambers for an identical period of time (10 min) without receiving the CS presentations. Water was not available within the chamber and the waterspout was not illuminated during the pre-exposure session.

#### Conditioning

Conditioning was conducted on the day following pre-exposure. No water was available within the chamber and the waterspout was not illuminated. There were two conditioning trials in which the UCS footshock was delivered following termination of the CS. The first pairing of CS and UCS was presented after 5 min had elapsed, and the second pairing was 5 min after the first, followed by a further 5 min left in the apparatus. In the absence of drinking, there were no behavioral measures to record.

#### Reshaping

On the day following conditioning, animals were reshaped following the same procedure as in pre-training sessions. This was in order to re-establish drinking after conditioning.

#### Test

On the day following reshaping, the animals were placed in the conditioning chambers and underwent an extinction test to the CS. Water was available throughout the test and the waterspout was illuminated. Once the animals had made 50 licks, the CS was presented for 15 min. The latency to make 50 licks in the absence of the CS (the A period) provided a measure of any individual variation in baseline lick responding. This was compared with the time taken to complete 50 licks following CS onset (B period) in a suppression ratio (A/(A+B)) to assess the level of conditioning to the CS, adjusted for any individual variation in drink rate.

##### Design and analysis

There were six experimental groups run in a 3×2 independent factorial design with lesion placement (at levels sham, PL or IL) and pre-exposure (levels of non-pre-exposed (NPE), and pre-exposed (PE)) as between subject factors. Statistical analysis was performed using analysis of variance (ANOVA) with alpha set at *P*<0.05 for the rejection of the null hypothesis. In cases of statistically significant main effects and simple effects, LSD post hoc comparisons were performed to assess differences between groups. T-tests were used to explore differences between groups in neurotransmitter levels. The dependent variables were lick latencies and totals for the pre-training and reshaping tests and the A period and suppression ratio for the test of conditioning.

## Results

### Neurochemical

Quantification of the selectivity of the lesions by HPLC revealed that six animals (3 IL and 3 PL operated animals) showed suboptimal levels of dopaminergic depletion (<40%) and consequently these animals were excluded from subsequent behavioral and neurochemical analysis. Thus after these exclusions, there were 20 sham-operated animals (10 PE, 10 NPE), 17 IL-lesioned animals (8 PE, 9 NPE) and 17 PL-lesioned animals (8 PE, 9 NPE).

Table 1 displays the levels (pmoles/μg protein) of DA, NA and 5-HT in the seven brain regions from which samples were taken as (A) absolute levels and (B) as the percentage depletion relative to sham levels. As is clear from this table, 6-OHDA infusions into the PL cortex were neuroanatomically selective as they produced significant depletion in DA levels in the target structure (∼71%) but spared DA terminals in the more ventral IL cortex. Infusion of 6-OHDA into the IL cortex depleted DA in the IL cortex (∼75%) but also to a lesser extent produced DA cell loss in the PL cortex (∼52%), suggesting some spread of the toxin to more dorsal regions. Desipramine pre-treatment did afford some neurochemical selectivity but there was some NA loss in the PL cortex following both lesions. 5-HT levels in the mPFC were unaffected by the 6-OHDA infusions. Neither the PL nor IL 6-OHDA lesion had any effect on striatal DA function as there were no significant changes in DA levels in the CPu or in either accumbal subterritory. Similarly, the lesions did not result in any significant changes in DA levels in the OFC or amygdala.

### Behavioral

#### Pre-training

Lesioned rats drank with similar latencies and in similar volumes to the sham operated controls (Mean latency to lick (s) (±S.E.M): shams=12.1 (±1.8); PL=14.4 (±2.6); IL=15.0 (±1.8). Mean total licks (±S.E.M): shams=1583.2 (±82.9); PL=1624.4 (±119.3); IL=1433.2 (±83.8). Statistically, there was no difference between the groups at this stage. This was confirmed by analysis of both time (s) to first lick (max *F*_(1,48)_=1.95, *P*=0.17) and total amount drunk (max *F*_(1,48)_=1.65, *P*=0.2).

#### Reshaping

Analysis of the times (s) to first lick in the reshaping session following conditioning revealed no effects of group, lesion or an interaction between these factors (max *F*_(1,48)_=1.4, *P*=0.24). Nor were there any differences between the groups in the total number of licks in the reshaping session (max *F*_(1,48)_=1.96, *P*=0.15).

#### Test

None of the experimental groups differed in the time to make the first lick in the absence of the noise (A period) in the test (max *F*_(2,48)_=1.65, *P*=0.2).

**Fig. 1:**
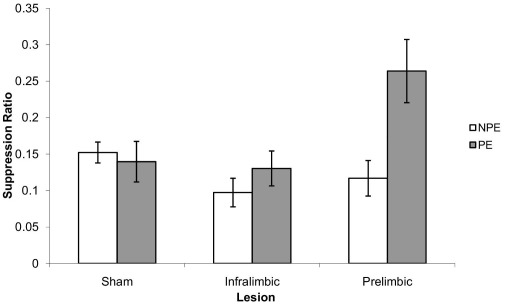
Mean suppression ratio (±S.EM) to the CS for non-preexposed (NPE) (white bars) and preexposed (PE) (light grey) groups following sham or 6-OHDA lesions to either the prelimbic or infralimbic mPFC.

The mean suppression ratios to the CS in the extinction test are presented in Fig. 1. Inspection of the figure reveals that, as expected under conditions of weak pre-exposure, there was no evidence of LI (i.e. reduced learning about the pre-exposed CS) in sham-operated animals. Similarly, the IL-lesioned group failed to show LI. However, there was a clear LI effect in the PL lesion group as there was markedly less suppression in the PE compared to the NPE group. This description of the data was supported by ANOVA which revealed a main effect of pre-exposure (*F*_(1,48)_=6.28, *P*<0.05), lesion (*F*_(2,48)_=3.77, *P*<0.05) but also a pre-exposure by lesion (*F*_(2,48)_=4.56, *P*<0.05) interaction. This interaction arose because there was no effect of stimulus pre-exposure in either sham- or IL-lesioned animals (both *F*′*s*<1) but robust LI in the PL group (*F*_(1,48)_=14.71, *P*<0.001). However, there were no differences between the lesion groups in terms of conditioning to the CS in the NPE condition (*F*<1).

## Discussion

The current experiment investigated the effects of DA depletion within the PL and IL mPFC on LI under experimental parameters (10 pre-exposures) designed to prevent the emergence of LI in sham-operated controls. Consistent with previous reports, under these experimental conditions sham-operated controls did not show LI and conditioned to the PE stimulus at equivalent levels to the NPE stimulus. However, 6-OHDA lesions to PL but not IL mPFC appeared to impair rats' ability to shift responding to the changed stimulus-reinforcement contingency at conditioning as these rats continued to respond according to the stimulus-no event acquired at pre-exposure. These results provide the first empirical demonstration of PL involvement in LI.

### Neuroanatomical and neurochemical specificity of the lesion

The 6-OHDA lesions to the PL were anatomically highly selective and produced no significant changes in DA in the more ventral IL cortex. In line with previous reports (e.g. Naneix et al., 2009), infusion of 6-OHDA into the IL led to DA denervation in both the target structure and more dorsally in the PL. Neither the PL nor IL 6-OHDA lesion had any effect on striatal DA as there were no significant changes in DA levels in the CPu or in either accumbal subterritory. This may at first seem surprising in view of evidence suggesting that mPFC lesions can increase the responsiveness of NAc DA (e.g. Deutch et al., 1990) but the current results are consistent with previous findings demonstrating that 6-OHDA lesions do not result in significant alterations in basal DA function within the ventral striatum (Rosin et al., 1992). Moreover, a lesion-induced increase in the responsiveness of NAc DA would be predicted to abolish rather than enhance LI (Joseph et al., 2000). Similarly, catecholamine functioning was also unaffected in the amygdala and OFC. This is important as the amygdala and OFC have both been implicated in the neural circuitry underpinning LI (e.g. Weiner, 2003) and hence the lack of changes in DA levels within these structures suggests that the behavioral effects seen at test are mediated by actions in the PL rather than in these structures. Despite desipramine pre-treatment, there was some evidence of changes in NA levels within the PL following lesions to both subregions. There is emerging evidence of interactive effects between DA and NA within the mPFC, and in particular the PL, which may account for this finding (Heidbreder and Groenewegen, 2003; Pan et al., 2004). Importantly, there was no evidence of non-specific neuronal damage as there were no significant changes in 5-HT levels in either prefrontal subregion.

### Comparisons to previous findings with mPFC lesions

Previously, excitotoxic lesions to the mPFC as well as local infusions of dopaminergic drugs have been found to be without effect on LI in paradigms designed to test for abolition of LI (e.g. Ellenbroek et al., 1996; Joel et al., 1997; Lacroix et al., 1998, 2000a,b). We would similarly expect the current lesions to spare LI using standard LI paradigms (i.e. high number of pre-exposures, low number of conditioning trials). However, in contrast to Schiller and Weiner (2004) lesions in the current study did lead to the emergence of LI under conditions that do not yield LI in controls. There are several potential explanations for the discrepancy between our findings and this previous report. Firstly the current study employed focal lesions that targeted the two main subregions of the mPFC, whereas the previous study was not anatomically selective. It is noteworthy in this respect that IL lesions in the current study, which depleted DA in both the IL and PL, were without effect on LI. Thus the results of the IL (plus PL) group here are consistent with findings that excitotoxic lesions of the entire mPFC do not potentiate LI (Schiller and Weiner, 2004). Such dissociable effects of anatomically selective and non-selective lesions within the mPFC mirror the demonstration that entire and subregionally-selective NAc lesions have dissociable and even opposing effects on LI (e.g. Gal et al., 2005). It remains to be seen what effect selective IL cortical lesions have on LI under conditions that do produce the phenomenon in sham-lesioned rats. Secondly, the current study employed lesions that targeted DA terminals within mPFC, while previously excitotoxic lesions that were not neurochemically selective were used. There is evidence that different neurochemical systems within the frontal cortex may regulate different aspects of cognitive control (e.g. Robbins and Roberts, 2007) and consequently the sensitivity of LI to manipulations to prefrontal function may depend on the neurochemical selectivity of the lesion.

Furthermore, there is evidence that the capacity of lesions to produce persistent LI depends on the experimental manipulation used to disrupt LI in shams (weak pre-exposure, context change, extended conditioning). For example, BLA lesions enhance LI with weak pre-exposure and extended conditioning but not with change in context (Schiller and Weiner, 2004, 2005). Hippocampal lesions, on the other hand, produce persistent LI with context change but not extended conditioning (Weiner, 2003). This dissociation has been attributed to the distinct roles played by the BLA and the hippocampus in reinforcement and contextual processes, respectively (Weiner, 2003). Such paradigm-dependent effects on LI may therefore account for the discrepancy between the results reported here and elsewhere. Indeed, the current results would suggest that PL involvement in LI is restricted to conditions of weak pre-exposure as previously mPFC lesions have been shown to be without effect on LI when the number of conditioning trials is increased (Schiller and Weiner, 2004). It remains to be established whether PL-lesioned animals are sensitive to changes in context between pre-exposure and conditioning. Taken together, the results from various lesion studies suggest that the involvement of particular brain structures (hippocampus, PL, OFC, BLA) in preventing the emergence of LI is specific to certain experimental conditions.

### PL involvement in LI

Since LI involves several processes (e.g. Lubow and Weiner, 2010), PL lesions could potentiate LI through a variety of mechanisms. Indeed, as the PL lesions in the current study were performed prior to behavioral training it is unclear whether PL function is critical to processes at pre-exposure, conditioning or test. According to the switching model of LI, LI involves the acquisition of two independent and competing associations at pre-exposure (CS-no event) and conditioning (CS-reinforcement) that compete to achieve behavioral expression (Weiner, 1990, 2003). The extent to which behavior is controlled by these two competing associations depends in part on the impact of conditioning, the strength of pre-exposure or the context. The absence of LI indicates switching of behavior to the CS-reinforcement association acquired at conditioning (Weiner, 2003; Weiner and Arad, 2009). The demonstration that lesions to the mPFC do not potentiate LI with extended conditioning (Schiller and Weiner, 2004) but mPFC and specifically PL lesions do enhance LI under conditions of weak pre-exposure suggests that the PL is not involved in modulating the impact of reinforcement on the expression of LI, a function that appears to be subserved by OFC and BLA (Schiller and Weiner, 2004). Rather, the emergence of LI despite limited pre-exposure indicates a failure to switch responding to the previously irrelevant but now relevant pre-exposed stimulus. This would suggest a role for the PL in integrating information about current stimulus-reinforcement contingencies and responding flexibly to changes in such contingencies. Thus in the intact brain and with limited CS pre-exposure, the PL may act to prevent the expression of LI by switching behavioral response strategies to the new reinforcement contingencies acquired at conditioning. However, as the strength of pre-exposure is increased by manipulating the number of pre-exposures, the role of the PL in re-attending behavioral responding to previously irrelevant stimuli that become relevant at conditioning may be overridden to allow the emergence of LI.

This proposition fits with considerable evidence demonstrating that lesions to mPFC cause cognitive inflexibility in a variety of behavioral paradigms (e.g. Ragozzino, 2007). In particular it appears that lesions to the mPFC disrupt animals' ability to respond adaptively when reinforcement contingencies are changed (e.g. Aggleton et al., 1995; Birrell and Brown, 2000; Bussey et al., 1997; de Bruin et al., 1994; Killcross and Coutureau, 2003; Ragozzino et al., 1999). This deficit tends to manifest as perseverative behavior as animals continue to respond according to previously acquired contingencies. These impairments reflect in part an inability to attend to cues that were previously irrelevant and to ignore previously salient cues that are no longer informative. Such a deficit would be predicted to produce LI under conditions that do not yield the phenomenon in controls, as PL lesioned-animals would fail to switch responding when the previously irrelevant (pre-exposed) stimulus becomes informative at conditioning. Moreover, there is good evidence for both DA and NA modulation of these processes. For example, blockade of D_1_ receptors (Ragozzino, 2002) and NA deafferentation (McGaughy et al., 2008) within the mPFC has been shown to disrupt rule shifting and prefrontal DA activity is increased during reversal learning (van der Meulen et al., 2007). Thus, the demonstration here that catecholamine depletion within the PL produces LI under conditions that do not yield it in controls is consistent with a role of prefrontal catecholamines in the regulation of adaptive and flexible behavior (Kehagia et al., 2010).

### Enhanced LI and schizophrenia

The current results highlight the importance of the use of appropriate experimental parameters in LI studies, as lesion effects on LI can be masked by procedures that are not suited to revealing potentiated (or abolished) LI (see Weiner and Arad, 2009). The demonstration of the disruptive effects of amphetamine on LI and the related DA hypothesis of the positive symptoms of schizophrenia has aroused considerable interest in the neural circuitry underpinning abolished LI as a model of the inability of schizophrenics to ignore irrelevant stimuli. However, it is becoming increasingly recognized that abnormally persistent LI under conditions that do not produce the phenomenon in controls may model certain aspects of the negative symptomology of schizophrenia as persistent LI may render the effect inflexible and unresponsive to situational demands (for reviews see Weiner, 2003; Weiner and Arad, 2009). The negative symptoms of schizophrenia are characterized by cognitive inflexibility and are associated with frontal-striatal dysfunction (e.g. Eisenberg and Berman, 2010). Significantly, it has recently been shown that LI is enhanced in chronic schizophrenic patients (Gal et al., 2009). The current findings suggest a modulatory role of catecholamines within the PL in the regulation of these processes.

## Figures and Tables

**Table 1A:** Mean absolute levels (±S.E.M) of DA, NA and 5-HT (pmoles/μg protein) of sham (pooled), PL- and IL-lesioned animals in PL, IL, OFC, NAc core and shell, CPu and amygdala

	DA			NA			5-HT		
	Sham	PL lesion	IL lesion	Sham	PL lesion	IL lesion	Sham	PL lesion	IL lesion
PL sample	0.07 (±0.011)	0.021 (±0.004)	0.031 (±0.007)	0.179 (±0.018)	0.065 (±0.011)	0.083 (±0.029)	0.126 (±0.014)	0.103 (±0.015)	0.118 (±0.028)
IL sample	0.115 (±0.016)	0.102 (±0.024)	0.028 (0.007)	0.378 (±0.039)	0.369 (±0.181)	0.366 (±0.229)	0.349 (±0.05)	0.215 (±0.036)	0.401 (±0.111)
OFC sample	0.784 (±0.203)	0.617 (±0.169)	0.693 (±0.183)	n.d.	n.d.	n.d.	0.128 (±0.014)	0.118 (±0.012)	0.108 (±0.011)
Core sample	9.22 (±1.01)	7.75 (±0.96)	9.71 (±1.24)	n.d.	n.d.	n.d.	0.352 (±0.043)	0.284 (±0.038)	0.379 (±0.06)
Shell sample	4.07 (±0.53)	3.81 (±0.59)	5.17 (±0.66)	1.02 (±0.159)	1.35 (±0.18)	1.20 (±0.164)	0.452 (±0.048)	0.442 (±0.056)	0.554 (±0.082)
CPu sample	7.17 (±0.44)	6.58 (±0.82)	7.04 (±0.61)	n.d.	n.d.	n.d.	0.168 (±0.027)	0.129 (±0.018)	0.174 (±0.033)
Amyg sample	0.393 (±0.097)	0.366 (±0.055)	0.319 (±0.066)	0.226 (±0.018)	0.226 (±0.024)	0.201 (±0.019)	0.323 (±0.032)	0.324 (±0.045)	0.295 (±0.03)

**Table 1B:** Mean percentage difference (±S.E.M) in DA, NA and 5-HT levels of PL- and IL-lesioned animals compared to PL and IL vehicle-infused sham animals in the seven brain regions assayed

	DA		NA		5-HT	
	PL lesion	IL lesion	PL lesion	IL lesion	PL lesion	IL lesion
PL sample	−71.3%⁎ (±6.5)	−52.3%⁎ (±11.5)	−66.9%⁎ (±6.7)	−55.7%⁎ (±16.5)	−16.3% (±11.9)	−7.9% (±21.9)
IL sample	−10.5%† (±21.2)	−75.4%⁎^,^† (±6.5)	−7.5% (±44.6)	−0.9% (±62.7)	−25.2% (±13.4)	+6.7% (±26.6)
OFC sample	−12.2% (±24.8)	−13.9% (±23.7)	n.d.	n.d.	−9.9% (±9.5)	−9.6% (±8.7)
Core sample	−13.1% (±10.6)	+1.3% (±8.7)	n.d.	n.d.	−29.2% (±10.2)	+13.4% (±22.3)
Shell sample	+3.8% (±33.7)	+24.9% (±36.0)	+30.1% (±21.1)	+23.4% (±16.4)	−0.7% (±12.4)	+21.3% (±17.8)
CPu sample	−4.1% (±10.4)	+0.9% (±8.6)	n.d.	n.d.	−28.8% (±9.9)	−4.1% (±18.1)
Amyg. sample	−7.1% (±13.9)	−19.0% (±16.8)	+0.1% (±10.6)	−11.3% (±8.9)	+0.5% (±13.9)	−8.2% (±9.4)

⁎ Significant difference from sham,

† significant difference from other lesion group, *P*<0.05, *t*-test.
